# Multi-Channel Power Scheduling Based on Intrusion Detection System Under DDoS Attack: A Starkberg Game Approach

**DOI:** 10.3390/s25030742

**Published:** 2025-01-26

**Authors:** Youwen Yi, Lianghong Peng

**Affiliations:** 1 Institute of Complexity Science, College of Automation, Qingdao University, Qingdao 266071, China; yiyouwen2023@163.com; 2 Shandong Key Laboratory of Industrial Control Technology, Qingdao 266071, China

**Keywords:** cyber-physical systems, Distributed Denial of Service (DDoS) attack, Intrusion Detection System (IDS), Starkberg game, optimal energy allocation

## Abstract

This study aims to explore the optimal power allocation problem under Distributed Denial of Service (DDoS) attack in wireless communication networks. The Starkberg Equilibrium (SE) framework is employed to analyze the strategic interactions between defenders and attacker under conditions of incomplete information. Considering the energy constraints of both sensors and attacker, this paper also proposes an Intrusion Detection System (IDS) based on remote estimation to achieve an optimal defense strategy, with Packet Reception Rate (PPR) serving as a criterion for intrusion detection. Targeting leaders and followers, the optimal power allocation solution is derived with Signal-to-Interference-Noise Ratio (SINR) and transmission cost as the objective functions. By combining the Adaptive Penalty Function (APF) method with the Differential Evolution (DE) algorithm, the study effectively addresses related non-linear and non-convex optimization problems. Finally, the effectiveness of the proposed method is verified through case studies.

## 1. Introduction

In recent years, cyber-physical systems (CPSs) have achieved fruitful results in the research field and have found extensive applications in domains including military defense, intelligent transportation, and smart grids [1,2,3,4,5]. However, due to the involvement of numerous critical infrastructures, any attack on CPSs can result in severe losses. With the emergence of various sophisticated intrusion techniques, attackers can disrupt network systems in a short period [6,7,8,9]. Major security incidents worldwide have highlighted the importance of CPS security issues. For example, in 2018, GitHub experienced 1.35 Tbps delayed traffic, leading to a 10 min service interruption; in September 2021, the Russian internet giant Yandex suffered a large-scale Distributed Denial of Service (DDoS) attack with requests per second reaching up to 21.8 million [10]. Therefore, with the rapid development of CPSs, the identification and prevention of network security intrusions have become particularly crucial.

In the literature [11,12,13], researchers have discussed the optimal power scheduling problem for Denial of Service (DoS) attacks based on Signal-to-Interference-plus-Noise Ratio (SINR) in CPSs, considering energy constraints of sensors and attackers. Li et al. [11] simulated the interaction decision-making process between sensors and attackers by establishing a Markov game framework. Ref. [12] proposed a Stackelberg Equilibrium (SE) framework to examine the strategic interaction between defenders and attackers in situations involving two distinct forms of incomplete information. Additionally, ref. [13] delved into a Stackelberg game involving a defender and multiple attackers. Distinguishing itself from the existing literature, which predominantly centers on equilibrium in static games, this study also reflects the dynamic process of Stackelberg games, demonstrating the intelligence of attackers in switching channel allocation for attack energy.

To ensure the security and reliability of network system resources, real-time monitoring of network transmissions is necessary to maintain confidentiality, integrity, and availability. The research in [14,15,16,17,18,19,20,21,22] proposes attack detection methods tailored to the constructed attack models, highlighting the existence of trade-off thresholds. In particular, the work in [19] designed a replay attack detection framework based on model-free reinforcement learning to effectively deal with attackers purposefully changing attack strategies. Furthermore, Agah et al. proved through a game theory framework that a Nash equilibrium is reached between attackers and Intrusion Detection System (IDS) [20]. In another study [22], a game-theoretic intrusion detection and defense method for DDoS attacks on the internet was proposed, modeling the interaction between the system and entities as a two-player Bayesian signal zero-sum game. These studies hold significant theoretical and practical value within the realm of network security, offering invaluable perspectives and solutions for real-time monitoring of network transmissions and the security of network system resources.

A Denial of Service (DoS) attack is a type of cyberattack where a single attacker floods a target system with excessive traffic or requests, aiming to overwhelm its resources and make it unavailable to legitimate users. The attack is typically launched from a single machine or a small number of machines [23,24,25,26,27,28]. A Distributed Denial of Service (DDoS) attack is a more advanced form of a DoS attack, where multiple compromised systems (often referred to as a “botnet”) are used to flood the target system with traffic. The distributed nature of the attack makes it much harder to defend against, as the traffic comes from many different sources, making it difficult to block all incoming connections without affecting legitimate users [6,29]. In most literature, Nash Equilibrium (NE) is commonly used to describe game situations where participants simultaneously choose actions. However, when decisions of defenders and attackers are made sequentially, the Stackelberg game framework is better suited to elucidate this procedure [12,30].

Although traditional DDoS attacks typically occur at the network layer, such as by flooding target servers with a large number of packets, physical layer attacks pose a significant threat in wireless sensor networks (WSNs). The open and interference-prone nature of wireless channels allows attackers to disrupt sensor node communications by transmitting high-power wireless signals, leading to degraded signal quality and even complete communication outages. Studies have shown that high-power interference can significantly reduce the signal-to-noise ratio (SINR) at the receiver, causing communication interruptions [31]. Additionally, physical layer attacks possess characteristics such as strong stealth and high energy efficiency, enabling attackers to achieve significant disruption with minimal energy expenditure [32]. To address this challenge, this study assumes that the attacker primarily influences system performance through physical layer interference. This assumption is supported by multiple studies that highlight the effectiveness of physical layer attacks in WSNs, especially under conditions of limited energy resources [33].

Unlike existing research, which typically assumes a static network environment and symmetric information between attackers and defenders [13,21], our study introduces dynamic channel changes, transmission costs, and the signal-to-interference-plus-noise ratio (SINR) into a Stackelberg game framework. This approach provides a more realistic simulation of real-world attack and defense scenarios. Additionally, our IDS uses remote state estimation for more precise detection, offering a clear advantage over traditional feature or anomaly-based methods. By considering energy constraints, environmental background noise, and time factors, we have developed a multi-stage Stackelberg game model that better reflects actual attack and defense interactions. These innovations significantly enhance system security and robustness, providing new theoretical and technical support for the field of wireless network security. The primary contributions of this research are outlined as follows:

1. Optimal energy scheduling under Distributed Denial of Service (DDoS) attack: The optimal energy scheduling problem is modeled in consideration of channels’ Signal-to-Interference-Noise Ratio (SINR) and transmission cost under Distributed Denial of Service (DDoS) attack. In the absence of an Intrusion Detection System (IDS), profit functions for the attacker and defenders are provided. To find the optimal solution, an improved differential evolution algorithm based on Adaptive Penalty Function (APF) is used to address the corresponding non-linear and non-convex optimization problems. Remote state estimation-based Detection System (IDS): We design an Intrusion Detection System (IDS) at the receiver end based on remote state estimation, using Packet Reception Rate (PPR) as the intrusion detection criterion. Unlike traditional feature- or anomaly-based methods [12,13], this approach is more suitable for real-world applications. Experimental results demonstrate that the presence of IDS can reduce the attacker’s profit and increase the defender’s profit. Multi-stage Stackelberg game model: A multi-stage Stackelberg game model is constructed to deal with the optimization problem, considering energy constraints of attacker and defenders, defenders’ awareness of environmental background noise, and introducing a time factor to build a finite-time Stackelberg game model. Compared to existing game models [12,13], our model better reflects the dynamic interactions between attackers and defenders, providing a more complex and realistic description of the attack and defense process. This new model offers valuable insights into developing effective defense strategies.

Notations: $R^{n}$ and $R^{m}$ represent the *n*-dimensional and *m*-dimensional Euclidean space. N and $N^{+}$ represent the sets of natural numbers and nonnegative integers, respectively. The superscript ′ denotes transpose. The symbols Pr(·) and E[·] represent the probability and expectation. $S_{+}^{n}$ is the aggregation of n×n positive semidefinite matrices. x·y is defined as $x^{\prime }y$ for vectors *x* and *y*. $1_{N}={\underset{N\;\mathrm{dimensional}}{\left\{\underset{︸}{1,1,\cdots ,1}\right\}}}^{\prime }$. ρ(·) presents the spectral radius of a matrix. For any two functions *f* and *g*, their composition is defined as (f∘g)(x)=f(g(x)).

## 2. Model Setup

### 2.1. Process and Sensor Model

**Figure 1:**
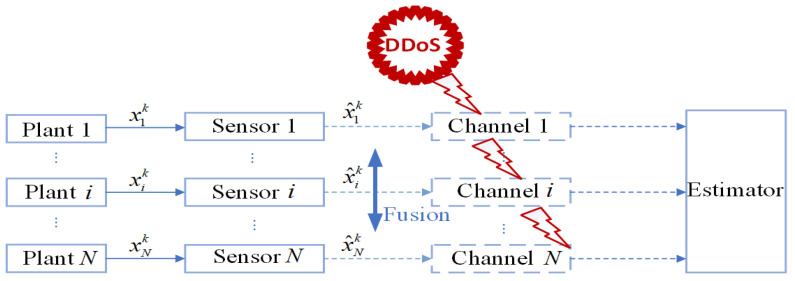
System model.

We consider discrete linear systems with multiple sensors as shown in Figure 1, consisting of a total of *N* independent discrete-time linear time-invariant systems and *N* sensors. The *i*-th sensor monitors the *i*-th system as follows:

(1) $$\begin{array}{cc} x_{i}^{k+1} & =A_{i}x_{i}^{k}+u_{i}^{k}\\ y_{i}^{k} & =C_{i}x_{i}^{k}+v_{i}^{k}, \end{array}$$

where i∈1,2,…N, the time index $k\in N^{+}$. $A_{i}\in R^{n_{i}\times n_{i}}$ is the state transition matrix, where $n_{i}$ is the dimension of the state vector $x_{i}^{k}\in R^{n_{i}}$. $C_{i}\in R^{m_{i}\times m_{i}}$ is the observation matrix, where $m_{i}$ is the dimension of the observation vector $y_{i}^{k}\in R^{m_{i}}$. $u_{i}^{k}\in R^{n_{i}}$ and $v_{i}^{k}\in R^{m_{i}}$ are independent zero-mean Gaussian white noises, satisfying $E\left[u_{i}^{k}u_{i}^{t}\right]=Q_{i}\in R^{n_{i}\times n_{i}}$, $E\left[v_{i}^{k}v_{i}^{t}\right]=R_{i}\in R^{m_{i}\times m_{i}}$, $E[u_{i}^{k}v_{i}^{t}]=0$, the covariance $Q_{i}\geq 0$, $R_{i}\geq 0$, $\forall t,\;k\in N^{+},\;i=1,2,\ldots ,N$. The initial state $x_{i}^{0}$ is a zero-mean Gaussian random vector with covariance $\Sigma_{i}^{0}\geq 0$, which is uncorrelated with $u_{i}^{k}$ and $v_{i}^{k}$. To avoid trivial issues, we assume that the system is unstable, i.e., $\rho (A_{i})>1$, i=1,2,…,N. We assume that $\left(A_{i},C_{i}\right)$ is observable, and $\left(A_{i},\sqrt{Q_{i}}\right)$ is controllable.

We assume that the sensors are “smart” and have sufficient computational capabilities. Following the measurement of the respective system at time step *k*, every sensor initiates a local Kalman filter to gauge the state of the process, incorporating all amassed measurements up to time *k* [34]. Subsequently, each sensor forwards its local estimate to a remote estimator. Based on the local estimate of the current state, we can calculate the minimum error estimate ${\hat{x}}_{i}^{k}$ of the local state of the *i*-th subsystem and the corresponding estimate error covariance matrix ${\hat{P}}_{i}^{k}$:

(2) $${\hat{x}}_{i}^{k}=E[x_{i}^{k}|y_{i}^{0},y_{i}^{1},\ldots ,y_{i}^{k}]$$

(3) $${\hat{P}}_{i}^{k}=E[\left(x_{i}^{k}-{\hat{x}}_{i}^{k}\right){\left(x_{i}^{k}-{\hat{x}}_{i}^{k}\right)}^{\prime }|y_{i}^{0},y_{i}^{1},\ldots ,y_{i}^{k}].$$

Computed by standard Kalman filtering [35]:

(4) $$\begin{array}{c} \left({\hat{x}}_{i}^{k},{\hat{P}}_{i}^{k}\right)=\mathrm{KF}\left({\hat{x}}_{i}^{k-1},{\hat{P}}_{i}^{k-1},y_{i}^{0},\ldots ,y_{i}^{k}\right). \end{array}$$

We further define the Lyapunov operator and Riccati operators $h_{i}$ and ${\tilde{g}}_{i}$: $S_{+}^{n}\to S_{+}^{n}$:

(5) $$\begin{array}{cc} h_{i}\left(X\right) & ≜A_{i}X{A_{i}}^{\prime }+Q_{i} \end{array}$$

(6) $$\begin{array}{cc} {\tilde{g}}_{i}\left(X\right) & ≜X-XC_{i}^{\prime }{\left[C_{i}XC_{i}^{\prime }+R_{i}\right]}^{-1}C_{i}X \end{array}$$

(7) $$\begin{array}{cc} h_{i}^{k}\left(X\right) & \overset{\Delta }{\;=}\underset{k\;\mathrm{times}}{\underset{︸}{h_{i}\circ h_{i}\ldots \circ h_{i}}}\left(X\right). \end{array}$$

where ∘ denotes the *k*-fold composition of the function $h_{i}$, i.e., the function $h_{i}$ applied consecutively *k* times. $X\in S_{+}^{n}$ is an n×n symmetric positive semi-definite matrix, representing the system’s covariance matrix or the estimation error covariance matrix.

The local estimate error covariance ${\hat{P}}_{i}^{k}$ converges to a steady-state value at an exponential rate. Therefore, we make the assumption:

(8) $$\begin{array}{c} {\hat{P}}_{i}^{k}=\bar{P_{i}},\;\forall k\geq 1,i\in 1,2,\ldots ,N, \end{array}$$

where $\bar{P_{i}}$ is the unique positive semi-definite solution to $\tilde{g_{i}}\circ h_{i}$.

### 2.2. Communication Model with SINR

After receiving the data packet, sensor *i* obtains a local estimate ${\hat{x}}_{i}^{k}$, which is then sent to the data fusion center through a wireless lossy channel. Due to channel attenuation and interference effects, random data loss occurs. To simulate this scenario, we assume that the channel uses an Additive White Gaussian Noise (AWGN) channel with Quadrature Amplitude Modulation (QAM) [11]. Subsequently, utilizing digital communication theory reveals the relationship between Symbol Error Rate (SER) and signal-to-noise ratio (SNR) [24]:

(9) $$\begin{array}{c} SER=2G\left(\varpi \sqrt{SNR}\right),\;G\left(x\right)≜\frac{1}{\sqrt{2\pi }}\int_{x}^{\infty }e^{-\frac{\varrho^{2}}{2}}d\varrho , \end{array}$$

where ϖ>0 is a parameter.

SNR can be described as:

(10) $$\begin{array}{c} \iota_{i}^{k}≜\frac{\zeta_{i}\theta_{i}^{k}}{\sigma_{i}^{2}+\sum_{j=1,j\neq i}^{N}H_{ij}\zeta_{j}\theta_{j}^{k}}, \end{array}$$

where $\zeta_{i}>0$ is the fading channel gain for channel *i*, $\sigma_{i}$ is the background noise, $\theta_{i}^{k}$ represents the transmission power allocated by sensor *i* to channel *i* at time *k*, $H_{ij}$ is the correlation coefficient between channels *i* and *j*.

We assume that DDoS attacks primarily occur at the physical layer, where the attacker interferes with sensor node communications by transmitting high-power wireless signals. If considering a DDoS attack on channel *i*, the SNR should be modified to the signal-to-interference-plus-noise ratio (SINR) as follows:

(11) $$\begin{array}{c} \mu_{i}^{k}≜\frac{\zeta_{i}\theta_{i}^{k}}{\sigma_{i}^{2}+\sum_{j=1,j\neq i}^{N}H_{ij}\zeta_{j}\theta_{j}^{k}+\phi_{i}\delta_{i}^{k}}, \end{array}$$

where $\phi_{i}>0$ is the fading channel gain for the attacker on channel *i*, and $\delta_{i}^{k}$ represents the transmission power allocated by the attacker to channel *i* at time *k*. Additionally, for sensors and attackers, the cost of transmitting unit power is assumed to be $\beta_{d}$ and $\beta_{a}$, respectively. In CPSs, wireless channels are susceptible to external interference. Attackers can significantly reduce the SINR at the receiver by increasing the transmission power $\delta_{i}^{k}$, thereby impacting the system’s performance.

### 2.3. DDoS Attack

Distributed Denial of Service (DDoS) attacks can block communication between components of cyber-physical systems (CPSs), thereby reducing the overall system performance. In current research, the attacker weakens system performance by continuously invading and maliciously disrupting communication channels. In real-life scenarios, energy constraints are an unavoidable issue for both sensors and attackers, impacting the performance of remote estimation and the strategies of both sides. Due to the presence of energy constraints, sensors need to manage energy efficiently to prolong their operational time, while attackers may exploit this limitation to design more destructive attack strategies. Therefore, when designing and deploying remote estimation systems, considering energy constraints is crucial for system stability and security. Attackers can use noise to interfere with communication channels between sensors and the fusion center.

In practical applications, both the defender and the attacker can estimate each other’s power levels by monitoring Channel State Information (CSI) or through other methods such as historical data analysis and predictive models [36,37]. Therefore, we assume that at time *k*, the defender knows the total power of the attacker is ${\bar{\delta }}^{k}$, and the attacker knows the total power of the defender is ${\bar{\theta }}^{k}.$ Here, ${\bar{\delta }}^{k}=\sum_{i=1}^{N}\delta_{i}^{k}$ represents the total power of the attacker across all channels at time *k*, while ${\bar{\theta }}^{k}=\sum_{i=1}^{N}\theta_{i}^{k}$ represents the total power of the defender across all channels at time *k*. On channel *i*, the total power of the attacker and the defender over the time horizon *T* is denoted as ${\bar{\delta }}_{i}$ and ${\bar{\theta }}_{i}$, respectively, where ${\bar{\delta }}_{i}=\sum_{k=1}^{T}\delta_{i}^{k},{\bar{\theta }}_{i}=\sum_{k=1}^{T}\theta_{i}^{k}$. Within the entire time range *T*, the total power of the attacker and the defender across all channels is denoted as $\bar{\delta }$ and $\bar{\theta }$, respectively, where $\bar{\delta }=\sum_{i=1}^{N}\sum_{k=1}^{T}\delta_{i}^{k},\bar{\theta }=\sum_{i=1}^{N}\sum_{k=1}^{T}\theta_{i}^{k}$.

In practical applications, attackers face energy budget constraints and therefore need to carefully decide whether to launch the DDoS attack on the wireless channel at each sampling instant. This strategic decision involves balancing the impact of the attack with energy expenditure. Attackers may formulate attack strategies based on system performance metrics, communication requirements, and their own energy resources. By effectively managing energy budgets and strategically timing attack, attackers can maximize the impact on the performance of remote estimators while ensuring their ability to sustain attack behavior. We use $\gamma_{i}^{k}=1$ to represent the attacker’s attack on channel *i* at time *k*; otherwise, $\gamma_{i}^{k}=0$. Within a finite time domain *T*, the *i*-th communication channel suffers a DDoS attack denoted by $\delta_{i}≜{\left\{\delta_{i}^{1},\delta_{i}^{2},\ldots ,\delta_{i}^{k},\ldots ,\delta_{i}^{T}\right\}}^{\prime }$, i=1,2,…,N. If $\delta_{i}^{k}>0$, then $\gamma_{i}^{k}=1$; if $\delta_{i}^{k}=0$, then $\gamma_{i}^{k}=0$. Within the time domain *T*, assuming the DDoS attacker applies constant interference power on the *i*-th communication channel, when the constant power $\delta_{i}^{k}$ is given, we can determine the total number of attacks on channel *i* within the time range *T* as $\gamma_{i}=\frac{{\bar{\delta }}_{i}}{\delta_{i}^{k}}$.

Therefore, the attacker’s attack strategy δ can be represented as:

(12) $$\begin{array}{c} \delta ≜\{\delta_{1},\delta_{2},\ldots ,\delta_{N}\}, \end{array}$$

The energy constraint faced by the attacker is:

(13) $$\begin{array}{c} \sum_{i=1}^{N}\sum_{k=1}^{T}\delta_{i}^{k}=\bar{\delta },\\ 0\leq \underset{min}{\delta }\leq \delta_{i}^{k}\leq \overset{max}{\delta }, \end{array}$$

where $\underset{min}{\delta }$ and $\overset{max}{\delta }$ represent the lower and upper power limits of $\delta_{i}^{k}$, respectively.

Similarly, we use $\vartheta_{i}^{k}=1$ to represent the *i*-th sensor selecting to transmit a data packet at time *k*; otherwise, $\vartheta_{i}^{k}=0$. Within a finite time domain *T*, the *i*-th sensor sends data packets denoted by $\theta_{i}≜{\left\{\theta_{i}^{1},\theta_{i}^{2},\ldots ,\theta_{i}^{k},\ldots ,\theta_{i}^{T}\right\}}^{\prime }$, i=1,2,…,N. If $\theta_{i}^{k}>0$, then $\vartheta_{i}^{k}=1$; if $\theta_{i}^{k}=0$, then $\vartheta_{i}^{k}=0$. Assuming the *i*-th sensor maintains a constant transmission power within the time domain *T*, when the constant power $\theta_{i}^{k}$ is given, we can determine the total number of data packets sent by the *i*-th sensor within the time range *T* as $\vartheta_{i}=\frac{{\bar{\theta }}_{i}}{\theta_{i}^{k}}$.

Therefore, the sensor’s transmission strategy θ can be represented as:

(14) $$\begin{array}{c} \theta ≜\{\theta_{1},\theta_{2},\ldots ,\theta_{N}\}, \end{array}$$

The energy constraint faced by the sensor is:

(15) $$\begin{array}{cc} & \sum_{i=1}^{N}\sum_{k=1}^{T}\theta_{i}^{k}=\bar{\theta },\\ & \underset{min}{\theta }\leq \theta_{i}^{k}\leq \overset{max}{\theta }, \end{array}$$

where $\underset{min}{\theta }$ and $\overset{max}{\theta }$ represent the lower and upper bounds of the transmission power $\theta_{i}^{k}$, respectively.

### 2.4. Remote Estimation with a Lossy Channel

In each time step *k*, sensor *i* transmits its result from the local Kalman filter ${\hat{x}}_{i}^{k}$ to a fusion center through a lossy communication channel. Let $\alpha_{i}^{k}\in \left\{0,1\right\}$ represent whether the data packet is received by the fusion center without errors. If it is successfully received, $\alpha_{i}^{k}=1$; otherwise, $\alpha_{i}^{k}=0$. When an attacker launches an attack and blocks the channel, sensor data packets are received with a probability $\omega_{i}^{k}$. Assume that $\alpha_{i}^{k}$ follows a Bernoulli distribution and

(16) $$\begin{array}{c} Pr\left(\alpha_{i}^{k}=1\right)=\omega_{i}^{k}. \end{array}$$

Using D(δ) to represent all the data packets received by the remote estimator at time step *k*, the remote estimator estimates the minimum mean square error ${\bar{x}}_{i}^{k}$ and covariance ${\bar{P}}_{i}^{k}\left(\delta \right)$ of the remote end based on the received D(δ), as follows:

(17) $$\begin{array}{cc} {\bar{x}}_{i}^{k} & =E[x_{i}^{k}|D\left(\delta \right)], \end{array}$$

(18) $$\begin{array}{cc} {\bar{P}}_{i}^{k} & =E[\left(x_{i}^{k}-{\bar{x}}_{i}^{k}\right){\left(x_{i}^{k}-{\bar{x}}_{i}^{k}\right)}^{\prime }|D\left(\delta \right)]. \end{array}$$

If ${\hat{x}}_{i}^{k}$ is successfully received, it is used to estimate ${\bar{x}}_{i}^{k}$; otherwise, the estimator forecasts the estimate using its prior estimate and the system model. Therefore, the minimum mean square error ${\bar{x}}_{i}^{k}$ and covariance ${\bar{P}}_{i}^{k}$ obtained by the remote estimator can be derived as follows:

(19) $$\begin{array}{c} \left({\bar{x}}_{i}^{k},{\bar{P}}_{i}^{k}\right)=\left\{\begin{array}{cc} ({\hat{x}}_{i}^{k},{\bar{P}}_{i}), & \;\mathrm{if}\;{\hat{x}}_{i}^{k}\;\mathrm{arrives},\\ A_{i}{\bar{x}}_{i}^{k-1},h_{i}\left({\bar{P}}_{i}^{k-1}\right), & \;\mathrm{otherwise}. \end{array}\right. \end{array}$$

At time *k*, ${\bar{P}}_{i}^{k}$ can only take values from a finite set $\left\{{\bar{P}}_{i},h_{i}\left({\bar{P}}_{i}\right),h_{i}^{2}\left({\bar{P}}_{i}\right),\ldots ,h_{i}^{k}\left({\bar{P}}_{i}\right)\right\}$, where $h_{i}\left({\bar{P}}_{i}\right)$ satisfies ${\bar{P}}_{i}\leq h_{i}\left({\bar{P}}_{i}\right)\leq h_{i}^{2}\left({\bar{P}}_{i}\right)\leq \ldots \leq h_{i}^{k}\left({\bar{P}}_{i}\right)$.

Therefore, we can represent the expected error covariance as:

(20) $$\begin{array}{c} E\left[{\bar{P}}_{i}^{k}\right]=\omega_{i}^{k}{\bar{P}}_{i}+\left(1-\omega_{i}^{k}\right)h_{i}\left(E\left[{\bar{P}}_{i}^{k-1}\right]\right). \end{array}$$

## 3. Payoff Functions

**Figure 2:**
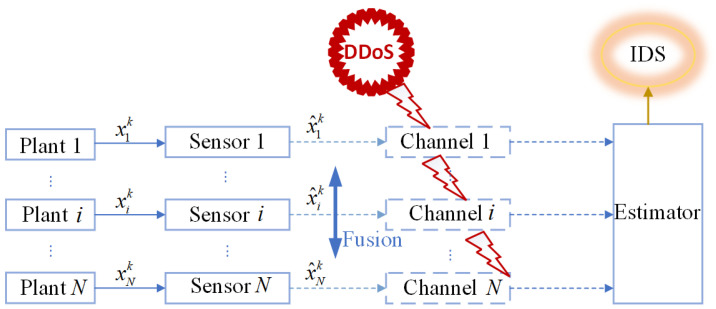
IDS model based on the remote estimator side.

This section will present the payoff functions of attackers and defenders in Figure 1 (Section 3.1), as well as the payoff functions of both parties with the presence of the Intrusion Detection System (IDS) detector in Figure 2 along with the corresponding constraint conditions (Section 3.2).

### 3.1. Rewards

We assume that the likelihood of the sensor perceiving solely background noise in the environment is denoted by $\varepsilon_{1}$. Therefore, the probability of a Intrusion Detection System (IDS) occurring is $\left(1-\varepsilon_{1}\right)$. The *N* systems we are considering are mutually independent, so we assume that the attack sequence for each system satisfies the following form:

$$\begin{array}{c} \left(0,\ldots ,0,\underset{\kappa_{i,1}}{\underset{︸}{1,\ldots ,1}},0,\ldots ,0,\underset{\kappa_{i,2}}{\underset{︸}{1,\ldots ,1}},0,\ldots ,0,\underset{\kappa_{i,\partial }}{\underset{︸}{1,\ldots ,1}},0,\ldots ,0\right) \end{array}$$

where 1 represents the attacker choosing to attack the channel at this moment, and 0 represents the attacker choosing not to attack at this moment, satisfying: $\sum_{s=1}^{\partial }\kappa_{i,s}=\gamma_{i}=\frac{{\bar{\delta }}_{i}}{\delta_{i}^{k}}$.

To evaluate the quality of estimation within the time interval *T*, we introduce the profit function of the attacker:

(21) $$\begin{array}{cc} O_{a}\left(\theta ,\delta \right)= & \frac{1}{T}\sum_{k=1}^{T}\left\{-\sum_{i=1}^{N}\frac{\zeta_{i}\theta_{i}^{k}}{\sigma^{2}+\sum_{j=1,j\neq i}^{N}H_{ij}\zeta_{j}\theta_{j}^{k}+\phi_{i}\delta_{i}^{k}}+\beta_{d}\sum_{i=1}^{N}\theta_{i}^{k}-\beta_{a}\sum_{i=1}^{N}\delta_{i}^{k}\right\}\\ = & \frac{1}{T}1_{T}\cdot \left\{-\left(1_{N}\cdot \mu \right)+\beta_{d}\left(1_{N}\cdot \theta \right)-\beta_{a}\left(1_{N}\cdot \delta \right)\right\}, \end{array}$$

where

$$\begin{array}{c} \mu_{i}^{k}={\left\{\mu_{i}^{1},\mu_{i}^{2},\ldots ,\mu_{i}^{T}\right\}}^{\prime },\;\mu =\left\{\mu_{1},\mu_{2},\ldots ,\mu_{N}\right\}, \end{array}$$

In (21), $-\beta_{a}\left(1_{N}\cdot \delta \right)$ represents the total power cost for the attacker. Additionally, introducing $\beta_{d}\left(1_{N}\cdot \theta \right)$ in the attacker’s reward function aims to deplete the defender’s energy.

Similarly, we assume that the transmission sequence for each sensor satisfies the following form:

$$\begin{array}{c} \left(\underset{\chi_{i,1}}{\underset{︸}{1,\ldots ,1}},0,\ldots ,0,\underset{\chi_{i,2}}{\underset{︸}{1,\ldots ,1}},0,\ldots ,0,\underset{\chi_{i,℘}}{\underset{︸}{1,\ldots ,1}}\right) \end{array}$$

The equation to satisfy is $\sum_{s=1}^{℘}\chi_{i,s}=\vartheta_{i}=\frac{{\bar{\theta }}_{i}}{\theta_{i}^{k}}$.

To evaluate the quality of estimation within the time interval *T*, we introduce the profit function of the defender:

(22) $$\begin{array}{cc} O_{d}\left(\theta ,\delta \right)= & \frac{1}{T}\sum_{k=1}^{T}\{\varepsilon_{1}\sum_{i=1}^{N}\frac{\zeta_{i}\theta_{i}^{k}}{\sigma^{2}+\sum_{j=1,j\neq i}^{N}H_{ij}\zeta_{j}\theta_{j}^{k}}-\beta_{d}\sum_{i=1}^{N}\theta_{i}^{k}\\ & +\left(1-\varepsilon_{1}\right)\sum_{i=1}^{N}\frac{\zeta_{i}\theta_{i}^{k}}{\sigma^{2}+\sum_{j=1,j\neq i}^{N}H_{ij}\zeta_{j}\theta_{j}^{k}+\phi_{i}\delta_{i}^{k}}\\ = & \frac{1}{T}1_{T}\cdot \left\{\varepsilon_{1}\left(1_{N}\cdot \iota \right)-\beta_{d}\left(1_{N}\cdot \theta \right)+\left(1-\varepsilon_{1}\right)\left(1_{N}\cdot \mu \right)\right\}, \end{array}$$

where

$$\begin{array}{c} \vartheta_{i}^{k}={\left\{\vartheta_{i}^{1},\vartheta_{i}^{2},\ldots ,\vartheta_{i}^{T}\right\}}^{\prime },\;\vartheta =\left\{\vartheta_{1},\vartheta_{2},\ldots ,\vartheta_{N}\right\}, \end{array}$$

In (22), $-\beta_{d}\left(1_{N}\cdot \theta \right)$ represents the total power cost for the defender.

### 3.2. Profit Function in the Presence of an IDS

In practical applications, when the estimator detects data loss due to unknown factors such as environmental changes or enemy intrusions, an Intrusion Detection System (IDS) is typically introduced at the receiving end, as shown in Figure 2, to effectively mitigate these influencing factors. This section introduces the concept of Packet Reception Rate (PRR) at the receiver end as a standard for intrusion detection. PRR refers to the ratio between successfully received packets by the estimator and the packets sent by the sensor. Therefore, $PRR_{i}$ can be expressed as:

(23) $$\begin{array}{c} PRR_{i}=\frac{{ℜ}_{i}}{k}, \end{array}$$

The parameter k represents the length of the time window, and the parameter ${ℜ}_{i}$ represents the number of packets received on the *i*-th channel within that time window.

**Remark 1.**  *When the packet reception rate is low, indicating a high packet loss rate, an Intrusion Detection System (IDS) may infer the presence of intruders in the system and issue an alert. To ensure the safe arrival of packets at the remote computer, sensors can adopt new technologies such as channel hopping. Channel hopping is a technique that dynamically changes channels during communication, enhancing the reliability and security of data transmission. Specifically, channel hopping technology switches between different channels at different time intervals, allowing packets to be transmitted through multiple channels. The benefit of this approach is that even if a specific channel experiences interference or packet loss, the system can still transmit data through other channels, improving the overall reliability of the communication link. By using channel hopping technology, sensors can select available channels to ensure that packets reach the remote computer safely, reduce the likelihood of packet loss, and enhance the performance and security of the system.*

We assume that when $PRR_{i}\leq PRR_{i}^{0}$, an alert will be triggered, where $PRR_{i}^{0}$ represents a predefined threshold value for triggering the alert.

**Lemma 1.**  *$PRR_{i}\leq PRR_{i}^{0}≜{\bar{P}}_{i}^{k}>h^{d_{i}}\left({\bar{P}}_{i}\right)$, k=1,2,…,T, where $d_{i}=k-{ℜ}_{i}^{0}$, ${ℜ}_{i}^{0}=max\left\{{ℜ}_{i}|\frac{{ℜ}_{i}}{k}\leq PRR_{i}^{0}\right\}$.*

**Proof.**  The triggering condition of the alarm $PRR_{i}\leq PRR_{i}^{0}$ is equivalent to ${ℜ}_{i}\leq {ℜ}_{i}^{0}$. Therefore, we assume that the number of packet losses $d_{i}^{num}$ for the *i*-th channel in any time window is less than or equal to $d_{i}^{num}\leq d_{i}=k-{ℜ}_{i}^{0}$, which leads to ${\bar{P}}_{i}^{k}\leq h^{d_{i}}\left({\bar{P}}_{i}\right)$. This contradicts the triggering condition $PRR_{i}\leq PRR_{i}^{0}$; thus, the assumption is not valid. By proof of contradiction, it is demonstrated that the triggering condition $PRR_{i}\leq PRR_{i}^{0}$ is also equivalent to $d_{i}^{num}>d_{i}=k-{ℜ}_{i}^{0}$.    □

From Lemma 1, it can be deduced that if the length of any attack sequence does not exceed $d_{i}$, the attack will not be detected by the detector. In fact, the attacker can steal the values of time window k and $PRR_{i}^{0}$ through the wireless channel, and then take action to carry out the attack. When the system has an IDS detector, the optimization of the payoff functions for the attacker and defender is as follows:

(24) $$\begin{array}{cc} O_{a_{IDS}}\left(\theta ,\delta \right)= & O_{a}\left(\theta ,\delta \right)-\frac{1}{T}\sum_{k=1}^{T}\sum_{i=1}^{N}\sum_{s=1}^{\partial }\left(d_{i}-\kappa_{i,s}\right)\frac{\zeta_{i}\theta_{i}^{k}}{\sigma^{2}+\sum_{j=1,j\neq i}^{N}H_{ij}\zeta_{j}\theta_{j}^{k}+\phi_{i}\delta_{i}^{k}}\\ = & \frac{1}{T}1_{T}\cdot \left\{-\left(1_{N}\cdot \mu \left[1_{s}\cdot \left(d-\kappa \right)\right]\right)+\beta_{d}\left(1_{N}\cdot \theta \right)-\beta_{a}\left(1_{N}\cdot \delta \right)\right\} \end{array}$$

(25) $$\begin{array}{cc} O_{d_{IDS}}\left(\theta ,\delta \right)= & O_{d}\left(\theta ,\delta \right)+\frac{1}{T}\sum_{k=1}^{T}\sum_{i=1}^{N}\sum_{s=1}^{\partial }\left(d_{i}-\kappa_{i,s}\right)\frac{\zeta_{i}\theta_{i}^{k}}{\sigma^{2}+\sum_{j=1,j\neq i}^{N}H_{ij}\zeta_{j}\theta_{j}^{k}+\phi_{i}\delta_{i}^{k}}\\ = & \frac{1}{T}1_{T}\cdot \{\varepsilon_{1}\left(1_{N}\cdot \iota \right)-\beta_{d}\left(1_{N}\cdot \theta \right)+\left(1-\varepsilon_{1}\right)\left(1_{N}\cdot \mu \left[1_{s}\cdot \left(d-\kappa \right)\right]\right), \end{array}$$

where

$$\begin{array}{cc} & \kappa_{i,s}=\kappa_{1},\kappa_{2},\ldots ,\kappa_{N};\;\kappa_{i}=\kappa_{i,1},\kappa_{i,2},\ldots ,\kappa_{i,\partial }\\ & d_{i}=d_{1},d_{2},\ldots ,d_{N}. \end{array}$$

In reality, the attacker can eavesdrop on the values of time ${ℜ}_{i}$ and k through wireless channels before taking any attack actions. In order not to be detected by the IDS, on the basis of the optimal attack strategy of the attacker in (13), a constraint condition is added:

(26) $$\begin{array}{c} \kappa_{i,s}\leq d_{i},\;i=1,2,\ldots ,N,\;s=1,2,\ldots ,\partial . \end{array}$$

Both the sensor and the attacker aim to maximize their own objective functions. Under the premise of energy constraints, the optimal strategies for both sides need to be found, and the main problem 1 needs to be solved.

**Problem 1.**  *The optimal attack schedule for the attacker:*

(27) $$\begin{array}{ccc} \; & \Omega_{a}^{*}\left(\delta \right)=argmax\;O_{a_{IDS}}\left(\theta ,\delta \right)\\ s.t.\; & \sum_{i=1}^{N}\sum_{k=1}^{T}\delta_{i}^{k}=\bar{\delta }, & \\ \; & 0\leq \underset{min}{\delta }\leq \delta_{i}^{k}\leq \overset{max}{\delta }, & \\ \; & \kappa_{i,s}\leq d_{i},\;i=1,2,\ldots ,N,\;k=1,2,\ldots ,T,\;s=1,2,\ldots ,\partial ; \end{array}$$

*and the optimal defense schedule for the defender:*

(28) $$\begin{array}{ccc} \; & \Omega_{d}^{*}\left(\theta \right)=argmax\;O_{d_{IDS}}\left(\theta ,\delta \right)\\ s.t.\; & \sum_{i=1}^{N}\sum_{k=1}^{T}\theta_{i}^{k}=\bar{\theta }, & \\ \; & 0\leq \underset{min}{\theta }\leq \theta_{i}^{k}\leq \overset{max}{\theta },\;i=1,2,\ldots ,N,\;k=1,2,\ldots ,T. \end{array}$$

## 4. Stackelberg Game for the Optimization Problem

The Stackelberg game is an important model in game theory. It describes a game process between a leader and a follower, where the defender, as the leader, formulates a strategy first, and the attacker reacts after observing the defender’s strategy. Both parties can formulate their own optimal strategies by analyzing the best response of the other party, and thus engage in a repeated game process, gradually approaching an equilibrium point. Under conditions of incomplete information, both parties need to infer the possible behaviors of the other party based on known information and probabilities, in order to formulate the optimal strategy. The elements of the Stackelberg game framework outlined in this paper are as follows:

- Players: *N* sensors and a Distributed Denial of Service (DDoS) attack.
- Strategy: The strategies of the defender and attacker are, respectively, $\theta ≜\{\theta_{1},\theta_{2},\ldots ,\theta_{N}\}$ and $\delta ≜\{\delta_{1},\delta_{2},\ldots ,\delta_{N}\}$.
- Reward: The reward function of the defender is $O_{d_{IDS}}\left(\theta ,\delta \right)$, and the reward function of the attacker is $O_{a_{IDS}}\left(\theta ,\delta \right)$.
- Interaction: A continuous-time two-stage dynamic game. First, the defender makes a decision as the leader, and then the attacker makes their own decision based on the defender’s decision, and so on.

We define the best responses of both sides in the game as follows [38].

**Definition 1.**  The best response is the action that brings the maximum return to a player while taking into account the actions of the other players. Specifically, the best responses for the defender and attacker are $\Omega_{d}^{*}\left(\theta \right)$ and $\Omega_{a}^{*}\left(\delta \right)$, respectively.

**Theorem 1.**  *The solution of the Stackelberg game first involves calculating:*

(29) $$\begin{array}{c} \theta^{SE}=\Omega_{d}^{*}\left(\Omega_{a}^{*}\left(\theta^{SE}\right)\right), \end{array}$$

*then computing*

(30) $$\begin{array}{c} \delta^{SE}=\Omega_{a}^{*}\left(\delta^{SE}\right), \end{array}$$

*Thus, $\left(\theta^{SE},\delta^{SE}\right)\in \left(\theta ,\delta \right)$ represents the equilibrium solution of the Stackelberg game.*

**Proof.**  When the defender’s defense strategy θ is given, the attacker chooses the attack strategy as $\delta =\Omega_{a}^{*}\left(\theta \right)$. The defender is aware of this reaction from the attacker, so the defender will choose a corresponding defense strategy to maximize their payoff $O_{d}\left(\theta ,\Omega_{a}^{*}\left(\theta \right)\right)$, which can be represented as:

(31) $$\begin{array}{c} \theta^{SE}=\Omega_{d}^{*}\left(\Omega_{a}^{*}\left(\theta^{SE}\right)\right). \end{array}$$

Upon observing the defender’s strategy $\theta^{SE}$, the attacker responds accordingly to determine the optimal strategy $\delta^{SE}$:

(32) $$\begin{array}{c} \delta^{SE}=\Omega_{a}^{*}\left(\delta^{SE}\right). \end{array}$$

Proof completed.    □

**Remark 2.**  *The Stackelberg equilibrium $\left(\theta^{SE},\delta^{SE}\right)$ and the Nash equilibrium $\left(\theta^{NE},\delta^{NE}\right)$ have significant differences. In the Stackelberg model, the leader first chooses the strategy $\theta^{SE}$, and then the follower selects the optimal response $\delta^{SE}$ based on the leader’s strategy. This sequential decision making mechanism allows the leader to optimize global performance, maximize the system’s total utility, and enhance security. In contrast, in the Nash equilibrium, all participants choose their strategies simultaneously, with each participant’s strategy considering only their own interests, which can lead to suboptimal solutions, especially in cases of conflict or competition. Additionally, the Stackelberg model typically offers better stability and predictability, making it suitable for long-term planning and global optimization scenarios. On the other hand, the Nash model may exhibit higher short-term utility in environments with high information transparency and ideal market conditions, but it tends to be less stable in dynamic and complex environments. The Stackelberg equilibrium must satisfy the following conditions:*

(33) $$\begin{array}{c} O_{d_{IDS}}\left(\theta ,\delta^{*}\right)\leq O_{d_{IDS}}\left(\theta^{*},\delta^{*}\right), \end{array}$$

(34) $$\begin{array}{c} O_{a_{IDS}}\left(\theta^{*},\delta \right)\leq O_{a_{IDS}}\left(\theta^{*},\delta^{*}\right). \end{array}$$

Considering the conditions that need to be met in problem 1, we design the attacker’s best response through the following optimization problem:

**Problem 2.** 
(35) $$\begin{array}{ccc} \; & \underset{\delta }{max}\;\frac{1}{T}1_{T}\cdot \left\{-\left(1_{N}\cdot \mu \left[1_{s}\cdot \left(d-\kappa \right)\right]\right)\right\}\; & \\ s.t.\; & \left(1_{N}\cdot \delta \right)-\bar{\delta }=0, & \\ \; & 0\leq \underset{min}{\delta }\leq \delta_{i}^{k}\leq \overset{max}{\delta }, & \\ \; & \kappa_{i,s}\leq d_{i},\;i=1,2,\ldots ,N,\;s=1,2,\ldots ,\partial , \end{array}$$

*which is equivalent to the following problem:*

**Problem 3.** 
(36) $$\begin{array}{ccc} \; & \underset{\delta }{min}\;1_{T}\cdot \left\{-\left(1_{N}\cdot \mu \left[1_{s}\cdot \left(d-\kappa \right)\right]\right)\right\} & \\ s.t.\; & \left(1_{N}\cdot \delta \right)-\bar{\delta }=0, & \\ \; & 0\leq \underset{min}{\delta }\leq \delta_{i}^{k}\leq \overset{max}{\delta }, & \\ \; & \kappa_{i,s}\leq d_{i},\;i=1,2,\ldots ,N,\;s=1,2,\ldots ,\partial . \end{array}$$

*Then, by calculation, we can obtain*

(37) $$\begin{array}{cc} & \frac{\partial 1_{T}\cdot \left\{\left(1_{N}\cdot \mu \right)\right\}}{\partial \delta_{i}^{k}}=-\left(1+d_{i}-\kappa_{i,s}\right)\frac{\zeta_{i}\theta_{i}^{k}\phi_{i}}{[\sigma^{2}+\sum_{j=1,j\neq i}^{N}\left(H_{ij}\zeta_{j}\theta_{j}^{k}+\phi_{i}\delta_{i}^{k}\right){]}^{2}} \end{array}$$

*and*

(38) $$\begin{array}{cc} & \frac{\partial^{2}1_{T}\cdot \left\{\left(1_{N}\cdot \mu \right)\right\}}{\partial {\delta_{i}^{k}}^{2}}=\left(1+d_{i}-\kappa_{i,s}\right)\frac{2\zeta_{i}\theta_{i}^{k}\phi_{i}^{2}}{[\sigma^{2}+\sum_{j=1,j\neq i}^{N}\left(H_{ij}\zeta_{j}\theta_{j}^{k}+\phi_{i}\delta_{i}^{k}\right){]}^{3}}. \end{array}$$

*The results indicate that when $\theta_{i}^{k}>0$ and $\kappa_{i,s}<d_{i}+1$, we have $\frac{\partial^{2}1_{T}\circ \{\left(1_{N}\circ \mu \right)}{\partial {\delta_{i}^{k}}^{2}}>0$. Clearly, for all $\forall \theta_{i}^{k}=0$, we have $\delta_{i}^{k}\left(\theta \right)=0$. The attacker can observe the defense strategy chosen by the defender; therefore, they only launch attacks upon detecting the transmission of sensor data to save energy. We assume that at time k, if i∈Ξ, then $\theta_{i}^{k}>0$; if $i\in \frac{N}{\Xi }$, then $\theta_{i}^{k}=0$, where Ξ=1,2,…,ψ, ψ denotes the number of $\theta_{i}^{k}>0$. Therefore, (36) can be solved through the following convex optimization problem:*

**Problem 4.** 
(39) $$\begin{array}{ccc} \; & \underset{\delta }{min}\;1_{T}\cdot \left\{-\left(1_{N}\cdot \mu \left[1_{s}\cdot \left(d-\kappa \right)\right]\right)\right\} & \\ s.t.\; & \left(1_{\psi }\cdot \delta \right)-\bar{\delta }=0, & \\ \; & \delta_{i}^{k}-\overset{max}{\delta }\leq 0, & \\ \; & -\delta_{i}^{k}+\underset{min}{\delta }\leq 0, & \\ \; & \kappa_{i,s}-d_{i}\leq 0,\;i=1,2,\ldots ,\psi ,\;s=1,2,\ldots ,\partial , \end{array}$$

*where*

(40) $$\begin{array}{c} \hat{\mu }=\left\{\mu_{1},\mu_{2},\ldots ,\mu_{\psi }\right\},\hat{\gamma }=\left\{\gamma_{1},\gamma_{2},\ldots ,\gamma_{\psi }\right\},\hat{\delta }=\left\{\delta_{1},\delta_{2},\ldots ,\delta_{\psi }\right\}. \end{array}$$

**Theorem 2.**  *If the defender’s strategy is θ, then the attacker’s best response can be calculated by the following formula:*

(41) $$\begin{array}{c} \delta_{i}^{k}\left(\theta \right)=\left\{\begin{array}{cc} max\left\{\frac{1}{\phi_{i}}\left[\sqrt{\left(1+d_{i}-\kappa_{i,s}\right)\frac{\zeta_{i}\theta_{i}^{k}\phi_{i}}{{\bar{\Phi }}_{i}^{k}}}-\sigma^{2}-\sum_{j=1,j\neq i}^{N}H_{ij}\zeta_{j}\theta_{j}^{k}\right],0\right\}, & i\in \Xi \\ 0, & i\in \frac{N}{\Xi } \end{array}\right. \end{array}$$

*where ${\bar{\Phi }}_{i}^{k}$ is obtained from the following equation:*

(42) $$\begin{array}{c} \sum_{k=1}^{T}\sum_{i=1}^{\psi }max\left\{\frac{1}{\phi_{i}}\left[\sqrt{\left(1+d_{i}-\kappa_{i,s}\right)\frac{\zeta_{i}\theta_{i}^{k}\phi_{i}}{{\bar{\Phi }}_{i}^{k}}}-\sigma^{2}-\sum_{j=1,j\neq i}^{N}H_{ij}\zeta_{j}\theta_{j}^{k}\right],0\right\}=\bar{\delta }. \end{array}$$

**Proof.**  We define the Lagrangian function at each time point *k* as:

(43) $$\begin{array}{cc} L(\hat{\delta },{ℶ}^{k},{ℸ}^{k},{ℑ}_{i}^{k},{ℷ}^{k})= & 1_{T}\cdot \left\{-\left(1_{N}\cdot \mu \left[1_{s}\cdot \left(d-\kappa \right)\right]\right)\right\}+\sum_{i=1}^{\psi }{ℶ}_{i}^{k}\left(\delta_{i}^{k}-\overset{max}{\delta }\right)\\ & +\sum_{i=1}^{\psi }{ℸ}_{i}^{k}\left(\delta_{i}^{k}+\underset{min}{\delta }\right)+\sum_{i=1}^{\psi }{ℑ}_{i}^{k}\left(\kappa_{i,s}-d_{i}\right)+{ℷ}_{0}^{k}\left[\left(1_{\psi }\cdot \delta \right)-\bar{\delta }\right], \end{array}$$

where ${ℷ}_{0}^{k}>0$, ${ℑ}_{i}^{k}\geq 0$, ${ℸ}_{0}^{k}\geq 0$, ${ℶ}_{i}^{k}\geq 0$, i=1,2,…,ψ. Thus, the Karush–Kuhn–Tucker (KKT) conditions can be represented as:

(44) $$\begin{array}{cc} \frac{\partial L(\hat{\delta },{ℶ}^{k},{ℸ}^{k})}{\partial \delta_{i}^{k}} & =0,\;i\in \Xi \end{array}$$

(45) $$\begin{array}{cc} \left(1_{\psi }\cdot \delta \right)-\bar{\delta } & =0 \end{array}$$

(46) $$\begin{array}{cc} {ℷ}_{0}^{k}\left[\left(1_{\psi }\cdot \delta \right)-\bar{\delta }\right] & =0 \end{array}$$

(47) $$\begin{array}{cc} {ℶ}_{i}^{k}\left(\delta_{i}^{k}-\overset{max}{\delta }\right) & =0,\;i\in \Xi \end{array}$$

(48) $$\begin{array}{cc} -{ℸ}_{i}^{k}\left(\delta_{i}^{k}+\underset{min}{\delta }\right) & =0,\;i\in \Xi \end{array}$$

(49) $$\begin{array}{cc} \sum_{i=1}^{\psi }{ℑ}_{i}^{k}\left(\kappa_{i,s}-d_{i}\right) & =0,\;i\in \Xi \end{array}$$

(50) $$\begin{array}{cc} \delta_{i}^{k}-\overset{max}{\delta } & \leq \;0 \end{array}$$

(51) $$\begin{array}{cc} -\delta_{i}^{k}+\underset{min}{\delta } & \leq 0,\;i\in \Xi \end{array}$$

(52) $$\begin{array}{cc} \kappa_{i,s}-d_{i} & \leq 0,\;i\in \Xi \end{array}$$

Through Equations (44) to (52), we systematically verify each part of the KKT conditions. Equation (44) ensures that the gradient of the objective function, when combined linearly with the Lagrange multipliers of all constraints, is zero, thereby satisfying the stationarity condition. Equations (45) to (49) ensure that all primal feasibility and dual feasibility conditions are met, including constraints such as energy limits and transmission power limits. Equations (50) to (52) utilize the complementary slackness conditions to determine which constraints are active, i.e., which constraints are binding at the optimal solution, where

(53) $$\begin{array}{cc} \frac{\partial L(\hat{\delta },{ℶ}^{k},{ℸ}^{k},{ℑ}_{i}^{k},{ℷ}^{k})}{\partial \delta_{i}^{k}}= & -\left(1+d_{i}-\kappa_{i,s}\right)\frac{\zeta_{i}\theta_{i}^{k}\phi_{i}}{[\sigma^{2}+\sum_{j=1,j\neq i}^{N}\left(H_{ij}\zeta_{j}\theta_{j}^{k}+\phi_{i}\delta_{i}^{k}\right){]}^{2}}\\ & +{ℷ}_{0}^{k}+{ℶ}_{i}^{k}+{ℶ}^{k}+{ℸ}^{k}+{ℑ}_{i}^{k}, \end{array}$$

Define ${\bar{\Phi }}_{i}^{k}={ℷ}_{0}^{k}+{ℶ}_{i}^{k}+{ℶ}^{k}+{ℸ}^{k}+{ℑ}_{i}^{k}>0$. Therefore, the relationship between $\delta_{i}^{k}$ and θ is given by (41).    □

**Remark 3.**  *It should be noted that ${\bar{\Phi }}_{i}^{k}$ is crucial for designing the optimal strategy $\delta_{i}^{k}\left(\theta \right)$ and the parameters ${\bar{\Phi }}_{i}^{k}$ should be computed first. However, since ${\bar{\Phi }}_{i}^{k}$ is not easily computable, we provide Algorithm 1 to solve for it.*

**Algorithm 1** Calculating parameter ${\bar{\Phi }}_{i}^{k}$
1:Calculate ${\bar{\Phi }}_{i}^{k}=\frac{\zeta_{i}\theta_{i}^{k}\phi_{i}}{[\sigma^{2}+\sum_{j=1,j\neq i}^{N}H_{ij}\zeta_{j}\theta_{j}^{k}{]}^{2}},i\in \Xi$2:Sort ${\bar{\Phi }}_{i}^{k}$: $0\leq {\bar{\Phi }}_{i^{1}}^{k}\leq {\bar{\Phi }}_{i^{2}}^{k}\leq \cdots \leq {\bar{\Phi }}_{i^{\psi }}^{k}$3:Find ${\bar{\Phi }}_{i}^{k}\in \left[{\bar{\Phi }}_{i^{h-1}}^{k},{\bar{\Phi }}_{i^{h}}^{k}\right]$ such that $f\left({\bar{\Phi }}_{i^{h}}^{k}\right)\leq \bar{\delta }\leq f\left({\bar{\Phi }}_{i^{h-1}}^{k}\right)$, where $f\left(\bar{\Phi }\right)=\sum_{k=1}^{T}\sum_{w=h}^{\psi }\frac{1}{\phi_{i^{w}}}\left[\sqrt{\frac{\zeta_{i^{w}}\theta_{i^{w}}^{k}\phi_{i^{w}}}{\bar{\Phi }}}-\sigma^{2}-\sum_{w=h,w\neq h}^{\psi }H_{i^{w}j}\zeta_{j^{w}}\theta_{j^{w}}^{k}\right]$4:if $f\left(\bar{\Phi }\right)=\bar{\delta }$, then ${\bar{\Phi }}_{i}^{k}=\bar{\Phi }$


The defender’s best response can be obtained by solving the following optimization problem:

(54) $$\begin{array}{ccc} \; & max\;G(\iota (\theta ),\mu (\theta )) & \\ s.t.\; & \left(1_{N}\cdot \theta \right)-\bar{\theta }=0, & \\ \; & 0\leq \underset{min}{\theta }\leq \theta_{i}^{k}\leq \overset{max}{\theta }.\;i=1,2,\ldots ,N,\;k=1,2,\ldots ,T, \end{array}$$

where $G\left(\iota \left(\theta \right),\mu \left(\theta \right)\right)=1_{T}\cdot \left\{\varepsilon_{1}\left(1_{N}\cdot \iota \left(\theta \right)\right)+\left(1-\varepsilon_{1}\right)\left(1_{N}\cdot \mu \left(\theta \right)\left[1_{s}\cdot \left(d-\kappa \right)\right]\right)\right\}$.

**Theorem 3.**  *If $\varepsilon_{1}\in \left(0,1\right)$ is given, then there exists $\theta^{SE}\in \theta$, so the defender’s best strategy can be $\theta^{SE}$. After obtaining $\theta^{SE}$, Algorithm 2 can be used to calculate $\delta^{SE}\in \delta$. Therefore, $\left(\theta^{SE},\delta^{SE}\right)$ is the Stackelberg equilibrium.*

**Algorithm 2** APF-based Differential Evolution
1:Construct adaptive penalty function:

Θ(θ)=Δ(θ)+η(θ)Λ(θ)

where $\Delta \left(\theta \right)=1_{T}\circ \left\{\varepsilon_{1}\left(1_{N}\circ \iota \left(\theta \right)\right)+\left(1-\varepsilon_{1}\right)\left(1_{N}\circ \mu \left(\theta \right)\left[1_{s}\circ \left(d-\kappa \right)\right]\right)\right\}$, $\eta \left(\theta \right)=1+\frac{\Delta (\theta )}{1+\Lambda (\theta )}$, $\Lambda \left(\theta \right)=\left(1_{N}\circ \theta \right)-\bar{\theta }$2:**Initialize:** $\theta_{i}^{k}\left(G\right)$ represents an N-dimensional variable, where $i=1,2,\ldots ,N_{p}$ and *G* is the generation. Choose $\theta_{j,i}^{k}\left(0\right)=\theta_{j_{min}}^{k}+rand\left(0,1\right)\left(\theta_{j_{max}}^{k}-\theta_{j_{min}}^{k}\right)$, where $i=1,2,\ldots ,N_{p}$, j=1,2,…,N and $\theta_{j_{min}}^{k}\leq \theta_{j}^{k}\leq \theta_{j_{max}}^{k}$.3:**Variation:** Generate variogram vector, as follows: $\delta_{i}^{k}\left(G\right)=\theta_{z_{1}}^{k}\left(G\right)+F\left(\theta_{z_{2}}^{k}\left(G\right)-\theta_{z_{3}}^{k}\left(G\right)\right)$, where $z_{1},z_{2},z_{3}\in 1,2,\ldots ,N_{p}$ and $z_{1}\neq z_{2}\neq z_{3}$, and the adaptive mutation operator *F* can be given by $F=F_{0}\times 2^{℧}$, where $℧=e^{1-\frac{G_{max}}{G_{max}+1-G}}$. $G_{max}$ represents the maximum generation.4:**Crossover:** Denote $\delta_{i}^{k}\left(G+1\right)=\left[\delta_{1,i}^{k}\left(G+1\right),\ldots ,\delta_{N,i}^{k}\left(G+1\right)\right]$, **If** $rand\left(0,1\right)\leq CR\;\;\mathrm{or}\;\;j=j_{rand}$,     $\delta_{j,i}^{k}\left(G+1\right)=\delta_{j,i}^{k}\left(G+1\right)$

**else**
     $\delta_{j,i}^{k}\left(G+1\right)=\theta_{j,i}^{k}\left(G+1\right)$where CR is crossover operator, and $j_{rand}\in \left\{1,2,\ldots ,N\right\}$ is a sequence selected randomly.


To obtain the defender’s strategy, the optimization problem (54) needs to be solved. However, due to the nonlinearity and possible non-convexity of the reward function $O_{d_{IDS}}\left(\theta ,\delta \left(\theta \right)\right)$, solving (54) is not easy. This paper combines the Adaptive Penalty Function (APF) method and the Differential Evolution Algorithm to handle related non-convex and nonlinear optimization problems [39]. Algorithm 2 details the main steps of the Differential Evolution algorithm based on APF. Differential Evolution (DE) is an efficient global optimization algorithm suitable for nonlinear, non-convex, and multimodal problems. Artificial Potential Field (APF) is a path-planning method that quickly finds feasible paths using potential fields. By combining DE and APF, we leverage DE’s global search capabilities to find the global optimum and APF’s local guidance to speed up convergence. This integration enhances optimization efficiency and better handles complex environments and constraints. Once $\theta^{SE}$ is obtained, the attacker’s best response $\delta^{SE}$ can be calculated according to Theorem 2.

## 5. Simulations

In this section, we use numerical examples to demonstrate the theoretical results of the Stackelberg game strategies under the presence of Intrusion Detection System (IDS) detectors as proposed in this paper. We set the following parameters: N=3, T=1000, ζ=[0.2;0.3;0.3], $\phi =\left[0.3;0.2;0.2\right],\;\sigma^{2}=0.02,\;\beta_{d}=0.2,\;\beta_{a}=0.2,\;0\leq \theta_{j}^{k}\leq 5,\;H_{12}=0.01,\;H_{13}=0.03,$ $H_{21}=0.02,\;H_{23}=0.01,\;H_{31}=0.01,\;H_{32}=0.02,\;N_{p}=50,\;G=100,\;F=0.4,\;CR=0.3,$ $PRR_{1}^{0}=PRR_{2}^{0}=PRR_{3}^{0}=PRR^{0}$. To further validate the proposed framework’s effectiveness in practical wireless communication, we refer to recent experiments using real-world datasets and testbeds. For instance, ref. [40] evaluated a similar strategy on an IEEE 802.11-based testbed, showing improved throughput and SINR under interference. Similarly, ref. [41] used USRP devices to simulate a multi-user environment and showed effective attack mitigation while maintaining high communication quality. These studies demonstrate advantages and achievements from different perspectives, providing useful references. It is worth noting that our proposed framework includes an IDS detector. Compared with [12], its uniqueness is introducing this detector at the estimator end. This innovation makes our framework more effective in practical scenarios. Through in-depth analyses, we find it more robust and applicable, offering more reliable guarantees and better performance.

**Figure 3:**
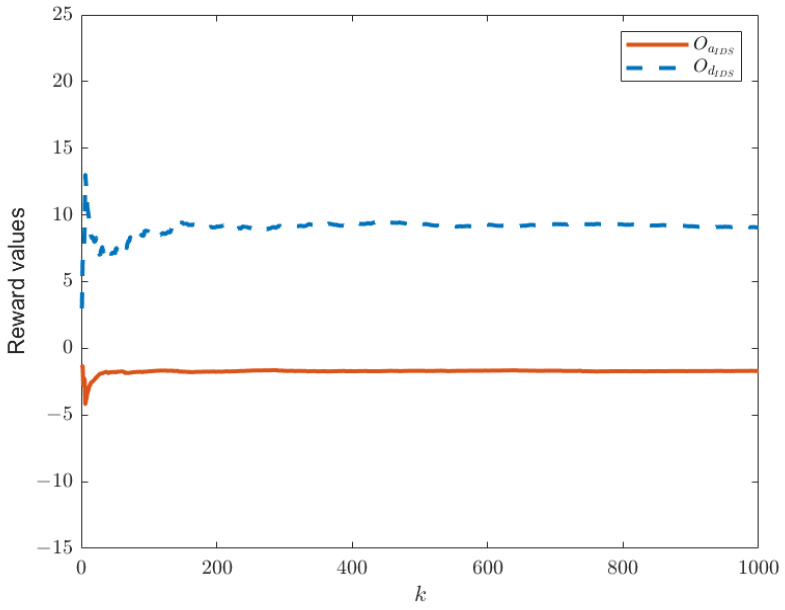
The optimal values of $O_{d}\left(\theta ,\delta \right)$ and $O_{a}\left(\theta ,\delta \right)$ in the absence of IDS.

**Figure 4:**
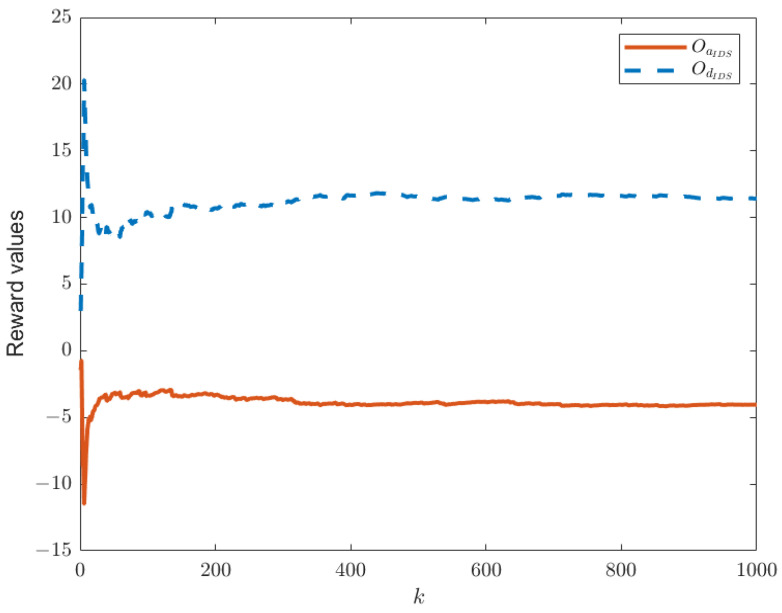
The optimal values of $O_{d_{IDS}}\left(\theta ,\delta \right)$ and $O_{a_{IDS}}\left(\theta ,\delta \right)$ in the presence of IDS.

**Figure 5:**
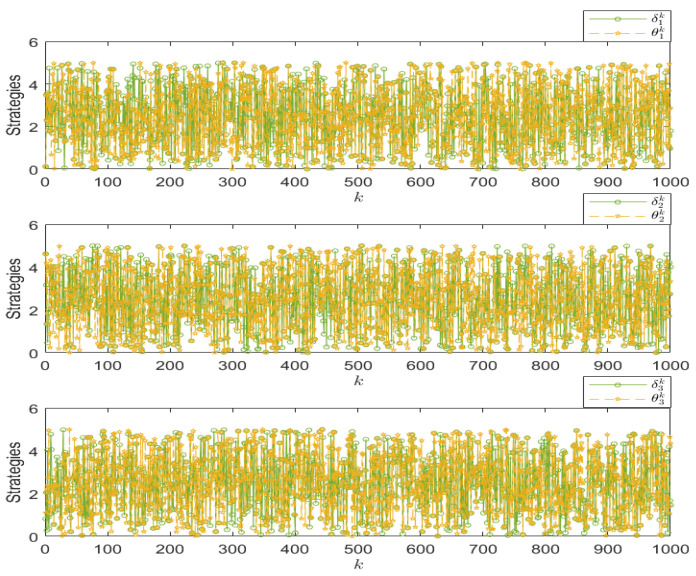
The optimal strategy in the presence of IDS.

When the IDS does not exist in the system model, as shown in Figure 1, the reward functions of both sides are as given by Equations (21) and (22). Keeping other parameters constant, we can establish the relationship between time *k* and the reward functions of both sides, as shown in Figure 3. In this paper, we have designed an IDS at the remote estimator end, as depicted in Figure 2. By choosing $\varepsilon_{1}=0.6$ and $PRR^{0}=5$, we are able to obtain the relationship between time *k* and the reward functions of both sides, as shown in Figure 4, as well as the strategic choices of both participants when reaching equilibrium in Figure 5. The three graphs from top to bottom in Figure 5, respectively, display the strategy values of attackers and defenders on channels 1, 2, and 3. By comparing Figure 3 and Figure 4, it can be observed that the proposed IDS detector in this paper can reduce the reward value of attackers and effectively increase the reward value of defenders. In Figure 4, after each participant undergoes 650 iterations, the reward function converges to the optimal value.

**Figure 6:**
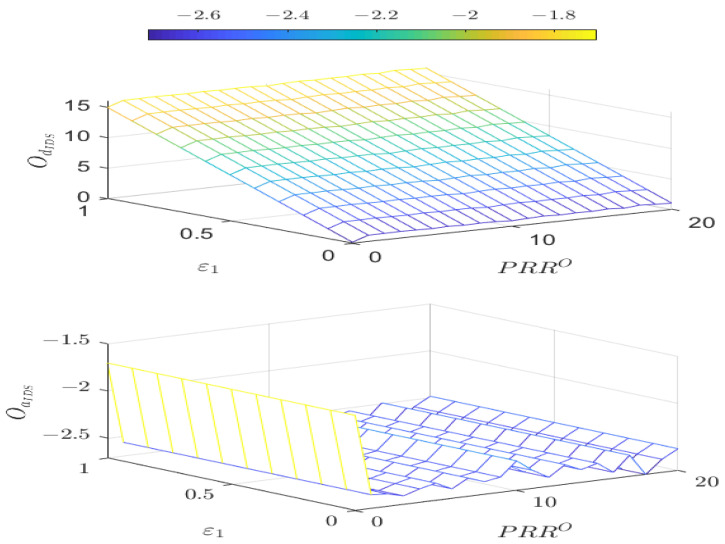
Reward functions $O_{d_{IDS}}\left(\theta ,\delta \right)$ and $O_{a_{IDS}}\left(\theta ,\delta \right)$ under different $PRR^{0}$ and $\varepsilon_{1}$.

By solving Theorem 2 and Algorithm 2, the relationship between the boundary values $PRR^{0}$, $\varepsilon_{1}$ triggering alarms in the IDS detector, and the reward values of both players is illustrated in Figure 6. We choose $PRR^{0}\in \left[0,10\right]$ and $\varepsilon_{1}\in \left[0,1\right]$. The first graph in Figure 6 represents the reward function of the defender, while the second graph illustrates the reward function of the attacker. From the graph, we can conclude that when $PRR^{0}$ is fixed, $\varepsilon_{1}$ has a significant impact on $O_{d_{IDS}}\left(\theta ,\delta \right)$, with an increase in $\varepsilon_{1}$ corresponding to a larger $O_{d_{IDS}}\left(\theta ,\delta \right)$. The influence of $\varepsilon_{1}$ on $O_{a_{IDS}}\left(\theta ,\delta \right)$ is minimal. When $\varepsilon_{1}$ is determined, changes in $PRR^{0}$ result in different reward values for both players in the game. As $PRR_{i}^{0}$ transitions from 0 to 1, it is evident that the reward function value of the attacker sharply decreases, while the defender’s reward value slightly increases. For $PRR_{i}^{0}\geq 1$ the attacker’s reward value exhibits minor periodic fluctuations, while the defender’s reward value remains relatively stable.

**Figure 7:**
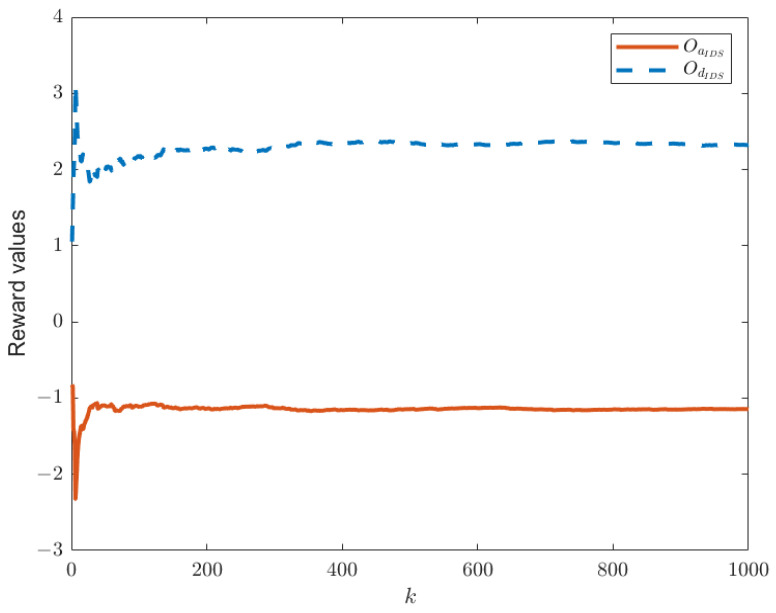
Strategies of the defender and attacker under $0\leq \theta_{j}^{k}\leq 0.5$.

In the simulation, we can select the range of values for $\theta_{j}^{k}$ based on the communication constraints of each channel, which in turn affects the optimization results. Therefore, the results obtained may not be the optimal solution but a suboptimal one. Given this, the nonlinear and non-convex optimization proposed in this paper can provide a feasible solution. In Figure 4, we have chosen $0\leq \theta_{j}^{k}\leq 5$. If we set $0\leq \theta_{j}^{k}\leq 0.5$, the reward function values for both parties are depicted in Figure 7.

Our optimization scheme combines the global search capabilities of Differential Evolution (DE) with the local guidance advantages of the Artificial Potential Field (APF) method. The time complexity of DE is $O(N_{p}\times G\times D)$, where $N_{p}$ is the population size, *G* is the maximum number of iterations, and *D* is the dimensionality of each individual. This complexity arises from the matrix operations involved in evaluating the fitness function. The time complexity of APF is $O(N^{2})$, primarily dependent on the number of nodes *N* and the size of the channel gain matrix *H*. Therefore, the overall time complexity of our combined approach is $O(N_{p}\times G\times \left(D+N^{2}\right)$. Based on our current experimental setup ($N=3,\;T=1000,\;N_{p}=50,\;G=100$), the average runtime is 2.5 s. Although these experiments were conducted on a smaller scale, we can extrapolate the performance for larger scales by adjusting parameters (e.g., $N=50,\;N_{p}=200,\;G=500$). Our complexity analysis suggests that while runtime will increase, it will remain within a reasonable range. To evaluate real-time performance, we simulated a scenario requiring one optimization decision per second. Under current settings, the optimization completes within 1 s, meeting real-time requirements. However, for larger problem scales, we may need to adjust parameters, such as reducing population size or lowering the number of iterations, to maintain real-time performance. We also explored parallel computing, where processing individuals across multiple nodes can significantly reduce runtime without sacrificing optimization effectiveness. This approach is a promising direction for future research.

## 6. Conclusions

This study aims to explore the optimal power control problem when the wireless channel undergoes Distributed Denial of Service (DDoS) attacks with the presence of an Intrusion Detection System at the remote estimator. Considering the impact of the attacker on the wireless channel, we quantify it using signal-to-noise ratio as a metric. To address this, we propose a Stackelberg game framework to analyze the game situation when both sides are in an incomplete information state. In such scenarios, the optimal strategy relationship between attackers and defenders can be determined through Karush–Kuhn–Tucker (KKT) conditions. We employ a Differential Evolution (DE) method based on Artificial Potential Field (APF) to design the optimal strategy for the defense side. Finally, we not only provide specific steps to solve the corresponding optimization problem but also observe that this optimization scheme can effectively reduce the reward function value for attackers and simultaneously increase the reward value for defenders when an Intrusion Detection System exists at the remote estimator. In future work, we plan to investigate integrated defense strategies that combine DDoS attacks with other network attacks (e.g., man-in-the-middle attacks, data tampering). By developing a multi-layered, multi-dimensional defense system, we aim to enhance overall security and improve the system’s resilience against complex attack environments.

## Data Availability

Data will be made available on request.
